# Conditional vs. Unconditional Quarantining for Acute Infectious Diseases Which Confer to Persons Having Them Immunity for Life*Read before the Aesculapian Club, January 20, 1910.

**Published:** 1915-02

**Authors:** Albert J. Colton

**Affiliations:** Physician to Buffalo Hospital Sisters of Charity and St. Mary’s Maternity Hospital; 27 Jewett Avenue


### Conditional vs. Unconditional Quarantining for Acute Infectious Diseases Which Confer to Persons Having Them Immunity for Life.*

ALBERT J. COLTON, M. D.,

Physician to Buffalo Hospital Sisters of Charity and St. Mary's Maternity Hospital.

By using the words conditional and unconditional I mean quarantining only when the disease has shown an unusual virulence or a mortality above a certain per cent. and not quarantining in every instance because a disease is infectious or contagious. We are all aware that the virulence of the same disease may differ in different epidemics, the mortality and complications running very high in some, while in others extremely low, the disease being so light that in many instances it is often unrecognized.

In this paper I shall refer only to those acute infectious diseases which occur in the individual but once, viz: scarlet fever, measles, chickenpox, mumps, whooping-cough and German measles. In this category I have purposely omitted small-pox because medical science has given to the world an artificial means of producing immunity, or at least lessening its severity and susceptibility, viz: vaccination. As to the other acute infectious diseases enumerated we know as little about producing an artificial immunity, or its specific cause today as we did one hundred years ago. This is due largely, I believe, to the fact of our inability to study these diseases in the lower animals, upon which the medical man has experimented so largely and produced such wonderful progress in many other diseases. In other words, we are unable to produce in the lower animals any of the acute infections mentioned, and are thus deprived of ascertaining and studying the specific germ (if it be one), which is the causative factor in the disease. That it is due to some form of micro organism, either animal or vegetable, can hardly be doubted.

In diphtheria, tuberculosis, tetanus, typhoid fever, rabies, yellow fever, malaria, cholera, and many other diseases wonderful strides have been made, the specific organism having been isolated, and in many instances are we able to produce immunity. The specific causes of these diseases have been determined largely by experimenting on the lower animals which could be inoculated with the disease itself in many instances; and apropos are not the benefits to mankind derived from the study of these diseases alone on the lower animals sufficiently great to justify any and all vivisection that has ever been done

*Read before the Aesculapian Club, January 20, 1910.

on them, notwithstanding the views of some of our brute loving antagonists, the anti-vivisectionists. Digressing a little more from our subject, would not the future study of these acute infections on criminals condemned to death or life sentences be justifiable and be a great aid to the solution of the specific cause of these diseases? To what better or more fitting purpose could they be put after years of crime to serve as a benefactor to their fellowman, thus, making partial atonement for their crimes.

The great desideratum in any and all diseases is to be able to produce immunity, and as the only way we have at present to do this in these acute infectious diseases is by having the disease itself, why not use natures means of immunity? Why then quarantine **except** in those epidemics which are of a severe type? We take chances in some of the other diseases to produce immunity, viz: to prevent smallpox we vaccinate every five years, and produce in many instances infections of a slight nature, like skin eruptions, callulitis, abscesses, and in rare instances more serious ones, erysipelas, tetanus, and death. We inject antitoxin in healthy patients at times to prevent diphtheria tetanus and rabies, and occasionally a death is produced.

The physician or surgeon urges his patient, and justly, recovering from frequent attacks of appendicitis that the appendix be removed, notwithstanding that he may never have another attack, and why? To produce immunity. Are we not taking chances when we try to produce these artificial forms of immunity? We certainly are, but they are so slight we are willing to take them. Why, then, when one of the acute infectious diseases mentioned exists in a mild form, should we postpone the disease by quarantining the family, and thus producing in many instances suffering, hardship and pauperism? It is among the poor that these hardships and sufferings are imposed, because it is among the poor that large families exist, and here it is that the disease is most likely to occur on account of the large size of the family and the poorer hygenic surroundings. The wage earner is compelled to quit work or leave home and pay his board, which, in many instances, is only enough to support his family, thus depriving them of that much when they are in sore need of more at this time. In many instances the family is compelled to call on charity, and thus have pauperism forced upon them. The children are forced to remain home, and on account of the diminutive size and scarcity of the rooms compelled to mingle with those that are sick, thus causing a spread of the disease instead of its suppression. The children are kept from school, first, because some one of them has chicken-pox, then later possibly measles, mumps, scarlet fever, etc., and are thus deprived of that greatest asset of civilized existence, an education.

I recently recall a case of measles in a poor family of several children where the father had tuberculosis and the mother went out daily to wash, scrub and iron to support the family, and which she did in a very plain, frugal way. The eldest son was having his last year in public school and doing excellent work, and the mother was making great sacrifices to have him finish, and was looking forward with pleasurable anticipations of having her son assist her in her daily burdens by means of an education to be received at the Technical High School which he was to enter the next year; but now things have changed; there is measles in the home. The mother is told not to leave the house. The boy must leave school, and is told by his principal that he will not be able to finish public school this year on account of it. The boy's aspirations to become a skilled mechanic or artisan at the Technical High School are now vanished. He must content himself by becoming a common drudge or laborer. Thus, a case of measles has entirely changed his future prospects. The children are compelled to stay at home huddled in one or two rooms with a tubercular father expectorating the death dealing germs with every cough. If they leave the premises some over zealous and disturbance making neighbor complains to the Board of Health, and a patrolman is probably stationed at the gate, and thus a family which were self sustaining and respectable are made paupers.

This is a common occurrence among the poor, and they just get over the measles and are again self supporting when another or the same one gets the mumps, chicken-pox or scarlet fever, and the drama is again repeated.

If by quarantining we expected to eventually stamp out or even indefinitely postpone these diseases then might we feel justified in the strictest quarantine, but under the present conditions this cannot even be hoped for. It seems to me simply a case of postponing the evil day from childhood to adult life when time and life is more valuable. Have we any proof that by postponing the disease today we are less apt to have it tomorrow, next year, decade, or far future, or that if we should contract the disease that it would be less severe? You say that statistics show that the adult is less susceptible; has it less severe when he does get it, and that the mortality is less. I say if statistics show this they are faulty and deceptive. The statistician argues that because a person has reached old age and never has been reported as having these diseases that he has avoided it when he was young by being quarantined, and when he became an adult was less susceptible. He does not take into consideration that a great many may have been naturally immune or less susceptible from their birth; that many have had the disease and it was never recognized, and others were never reported, either with the object of defeating the law or because it was the second, third or fourth case in the family at the quarantining stage of the first case, and not considered necessary to be reported.

That certain individuals have a natural immunity or a lessened susceptibility cannot be denied. Were it not so how would you account for an individual sleeping and remaining with another during a whole sickness and not contracting the disease? This I have seen and so have you. Of course there will not be as many cases among adults, because at least 95% have either had the disease or are naturally immune, and the other 5% have possibly avoided exposure until adult life, but always to their regret when they do get it.

Which is the more pathetic and serious to a family or community, to see a child take and possibly succumb to the disease early in life, or the head of the family, the wage earner, upon whom many may depend? I recall not long ago a nurse who had escaped scarlet fever until she was preparing herself for her life work at a hospital where she contracted the disease and died. I recall physicians who had dodged the disease during youth, but when they became of use to themselves and community, contracted the disease and narrowly escaped death, and others died. This is not an unusual thing. A person who has used all manner of means to dodge the disease in his youth and then contract it in his adult life at some inopportune time and place is like a person who has escaped a wild beast or enemy when armed, to at last meet his foe face to face bare handed.

What a continuous worry and annoyance to the person who has escaped the contagious disease to be constantly dodging it and at last meet it at some inopportune time and place when miles from home or friends. Then he always wishes that he had had it while young.

That a child under five years of age when adenoid and lymphoid tissue is more abundant about the nose or pharynx may have scarlet fever more severely I will not deny; but this is the age that the child is least apt to take it, because the child does not attend school, a common breeding place for infections, and it is at an age where the child can be best kept from those who have it. I also believe that whooping-cough is more severe in a child under five years of age, but for the same reason, they would be less apt to contract it.

Oft times on account of fear of being quarantined mild cases have been concealed, no physician being called, and only to be divulged when desquamation and a possible fatal nephritis has developed. A case of this kind I was called to see not long ago with another physician. Had not the family feared the ban of a yellow card a physician would have been called early and the fatal sequela intercepted. The mortality of measles is often increased by the disease being concealed and treated by the laity. The family knows that if the physician is called the case will be reported, the children will be kept from school; no laundry can be sent out, and a general inconvenience caused. The child is treated in the old traditional way, kept in a hot, dark room, with all the surplus bedding in the home piled upon it, with hot drinks, and the patient running a temperature of 104 or more. Every crack in the room is closed, even to the keyhole, lest a draft of cold air should come in and give the child pneumonia. The air becomes foul and putrid, with the proverbial measly smell. There is a catarrhal condition of the whole respiratory and mucus tract, and if ever a tubercular germ was left or admitted into the room it would find a most ideal breeding place in the respiratory tract of the patient. If the patient develops tuberculosis it is laid to the measles instead of the treatment.

Would not a more scientific way of handling the situation be to have all cases reported and immediately inspected, and if the hygienic surroundings and circumstances are unsatisfactory for treating a contagious disease have it removed to a first class contagious hospital where every scientific means for treating that disease could be had and handled only by specialists in that line, and thereby reducing the mortality to a minimum? The municipality could as economically care for the sick child in an up-to-date scientific hospital as to quarantine the whole family, thus making them a public charge.

Epidemics of these diseases, I believe, are produced by quarantining rather than prevented. If no quarantining were done we would have an even percentage of cases each year, but when quarantining is instituted the spread of the disease is held in check until a large percentage of the susceptible generation have escaped. At last some mild, unrecognized case gets into a school where none are immune, and its spread is like a fire in a wood shed; no stopping it until all have been made immune by having the disease. Then there will be a suspension of the disease for possibly 5 years or a decade and again ready for another outbreak.

Why keep trying to postpone the disease? Are we not better prepared to cope with the enemy when we know that it is in our midst, than to be taken unawares like a thief in the night? Instead of constantly locking our doors against the enemy, let us meet him in the open, and conquer him when we encounter him, and he will work out his own self destruction.

Why would it not be more humane, life-saving and justifiable to quarantine for tuberculosis, syphilis, gonorrhoea, influenza, and even itch and head lice, than for chicken-pox, mumps, measles and scarlet fever.

Certainly the mortality of tuberculosis is greater than any of the acute infections. We know the specific cause and better how to cope with it. Influenza is proven to be contagious and due to a specific organism, and productive of almost as much evil as scarlet fever. Pneumonia is frequently caused from the influenza bacillus as well as mastoid disease and meningitis. Syphilis and gonorrhoea have indirectly produced more deaths and heart-aches than scarlet fever ever did. These are diseases which by quarantining we could finally eradicate from the face of the earth, for we know the true and specific cause of the disease, and therefore how to fight it.

The great cry of closer inspection and quarantining for the acute infectious diseases when the type is mild and overlooking some of the more serious infections is in my mind simply "straining at a gnat and swallowing a camel."

I have written this paper with considerable timidity and hesitation, and expect some very severe criticisms, nevertheless they are my honest views from my own experience, and therefore have no apologies to offer, and again I reiterate that the unconditional quarantining for the acute infectious diseases, which confer to the individual having them immunity for life, is unjustifiable and simply postponing the evil day.

27 Jewett Avenue.

---

**Gluzinski Test for Gastric Ulcer and Cancer.** ("Med. Rev. of Revs.," Dec., 1914.) 1. Remove fasting contents if any. 2. Lavage. 3. Test breakfast of 1 boiled white of egg and 200 c.c. of water, recovered after $\frac{3}{4}$ hour. 4. Lavage. 5. Immediately, a test dinner of beef steak and 250 c.c. of water, recovered after $3\frac{3}{4}$ hours. In ulcer, free HCl is found after both meals, perhaps in the fasting contents. Even if present in the first contents, its absence or diminution in the second points toward cancer. (Note: All tests of this sort are fallacious. Excess of HCl is not especially characteristic of ulcer. Ewald's statistic study—though many of the individual series were imperfectly observed—shows approximately an even division between normal and abnormal states of acidity and the latter are about equally divided between excess and deficiency. Diminished HCl is characteristic of cancer, after it has become fairly well progressed but it is also characteristic of various other local and general conditions of a depressive nature. If the Gluzinski test is adopted merely as a guide to secretory tendencies and considered diagnostic of ulcer or cancer merely in a suggestive way, well and good. The trouble is, it will be taken as definitely diagnostic, exaggerated in value by early statistics based on cases already more or less definitely diagnosed, and then dismissed as worthless on the evidence of subsequent, contradictory statistics.)

---

**Sublingual Medication,** is advocated by Paulson, "Brit. Med. Jour.," especially for alkaloids and in military practice to save time.

